# Comparison of Fentanyl and Dexmedetomidine in Preventing an Increase in Heart Rate During Intubation Among Patients Undergoing General Anesthesia: A Meta-Analysis

**DOI:** 10.7759/cureus.26194

**Published:** 2022-06-22

**Authors:** Saniya Mohsin, Zubair Ahmad Ganaie, Hayan Kundi, Muhammad Bilal Ahmed, Bushra Riaz, Noman Khurshid Ahmed, Venkata Anirudh Chunchu, Areeba Haq

**Affiliations:** 1 Baqai Medical College, Baqai Medical University, Karachi, PAK; 2 Internal Medicine, Holy Family Red Crescent Medical College, Srinagar, IND; 3 Medicine, Fazaia Medical College, Karachi, PAK; 4 Medicine, Dow International Medical College, Karachi, PAK; 5 Radiology, Dow University of Health Sciences, Civil Hospital Karachi, Karachi, PAK; 6 Medicine, Dow University of Health Sciences, Karachi, PAK; 7 Medicine, Avalon University School of Medicine, Willemstad, CUW; 8 Critical Care Medicine, United Medical and Dental College, Karachi, PAK

**Keywords:** randomized control trial, heart rate, intubation, dexmedetomidine, fentanyl

## Abstract

General anesthesia induction, tracheal intubation, extubation, and laryngoscopy are associated with specific hemodynamic changes. Tracheal intubation and laryngoscopy are related to sympathetic stimulation and lead to hypertension and tachycardia. Recent studies have shown that dexmedetomidine is safe and effective as it does not depress respiratory function. This meta-analysis aims to compare the efficacy of dexmedetomidine and fentanyl in preventing an increase in heart rate (HR) during intubation among patients undergoing general anesthesia. A systematic literature search was done using PubMed, Cochrane Library, and Embase to assess studies comparing the efficacy of dexmedetomidine and fentanyl in preventing an increase in HR during intubation. A meta-analysis was done utilizing a random-effects model, and mean differences of HR were determined between fentanyl and dexmedetomidine at baseline, one minute, five minutes, and 10 minutes of intubation. In this meta-analysis, eight randomized control trials were included, involving 548 patients (274 in the fentanyl group and 274 in the dexmedetomidine group). The findings showed that significant difference of HR was significantly lower in the dexmedetomidine group than the fentanyl group at one minute of intubation (mean difference = -8.46; P-value = 0.003), at five minutes of intubation (mean difference = -7.51; P-value = 0.001), and at 10 minutes of intubation (mean difference = -5.15; P-value = 0.030). In the current meta-analysis, dexmedetomidine was better than fentanyl in preventing tachycardia following endotracheal intubation. HR was significantly lower at one minute, five minutes, and 10 minutes after intubation in the dexmedetomidine group compared to the fentanyl group.

## Introduction and background

General anesthesia induction, tracheal intubation, extubation, and laryngoscopy are associated with specific hemodynamic changes. Tracheal intubation and laryngoscopy are related to sympathetic stimulation and lead to hypertension and tachycardia [1]. These changes in hemodynamics may increase the risk of myocardial ischemia. As a result, effective blunting of these unpleasant responses is required [2]. Benzodiazepines, ketamine, propofol, remifentanil, fentanyl, and dexmedetomidine are all used to sedate patients during intubation [3,4]. Each of these drugs has certain disadvantages and advantages. For instance, propofol, opioids, and benzodiazepines, even though causing attenuating hemodynamic response and sedation, can lead to respiratory depression [5]. Recent studies have shown that dexmedetomidine is safe and effective as it does not depress respiratory function [6,7].

Compared to clonidine, dexmedetomidine is a newer two-receptor agonist with eight times better selectivity and affinity for the presynaptic alpha 1 receptor [8]. Dexmedetomidine, which has been utilized in infusion, for this reason, reduces these potentially dangerous cardiovascular effects during anesthetic induction [9]. Fentanyl is another agent with a short duration of action and quick onset. It can be used as a component to balance general anesthesia. Fentanyl reduces sympathetic outflow and mitigates the hemodynamic stress response via acting on opioid receptors [10].

There has been little investigation into comparing the efficacy of dexmedetomidine and fentanyl using multi-center large sample randomized controlled trials (RCTs). Furthermore, different studies yielded conflicting results. Thus, the current meta-analysis aimed to answer the question using a large sample size. This meta-analysis aims to compare the efficacy of dexmedetomidine and fentanyl in preventing an increase in heart rate (HR) during intubation among patients undergoing general anesthesia.

## Review

Materials and methods

Search Strategy

The current meta-analysis was carried out using the Preferred Reporting Items for Systematic Reviews and Meta-Analyses (PRISMA) guidelines. Two researchers independently carried out a systematic search of the published studies. No restrictions were placed on the place of publication. Electronic databases searched to find relevant articles included PubMed, Cochrane Library, and Embase. The keywords and MeSH (Medical Subject Headings) terms used to conduct the search included “dexmedetomidine,” “fentanyl,” “randomized controlled trial,” “adults,” “response,” “intubation,” and “heart rate.” Moreover, the references list of all selected articles was also searched to prevent any eligible articles from being missed out.

Study Inclusion Criteria

The trials used to conduct this meta-analysis included adult patients (aged 18 years or more) undergoing surgery who were randomly assigned into one of the two groups, i.e., fentanyl and dexmedetomidine. Studies involving children, including emergency surgical procedure patients, studies other than randomized controlled trials, studies published before 2015, and published in a language other than English were excluded from this meta-analysis.

Data Extraction

Two reviewers selected eligible studies independently, utilizing a standard data collection table for extracting data and recording the characteristics of trials. For each selected study, information collected included the name of the first author, date of publication, number of participants in each group, dose of drug received, and surgical procedure.

Outcome Measures

The primary outcome measures analyzed were HR at various points during surgery, including at baseline, one minute, five minutes, and 10 minutes of intubation.

Assessment of Risk of Bias

All eligible studies were read and evaluated by two reviewers independently to assess the methodological validity utilizing Cochrane Handbook version 5.0.2. Discrepancies were resolved via joint discussion, and a third researcher assisted in the decision, if necessary. Information evaluated for this purpose included blinding, random sequence generation, selective reporting, allocation concealment, and other kinds of biases. Each of these was graded as “low risk of bias,” “uncertain risk of bias,” and “high risk of bias.” This information was utilized for guiding our interpretation of the presented data table that was incorporated into the review findings interpretation through sensitivity analysis. The risk of bias graph describes all judgments and was drawn using RevMan software version 5.4.1 (The Cochrane Collaboration, Copenhagen, Denmark).

Statistical Analysis

The meta-analysis was carried out using RevMan software (version 5.4.1). The outcome of interest was HR. The treatment effect was estimated with a mean difference (MD) in the final values of HR between the fentanyl and dexmedetomidine groups. A comparison of final measurements in a randomized trial can normally be assumed to offer the same estimate as a comparison of changes from baseline. Statistic I^2^ was calculated to assess heterogeneity. For heterogeneity analysis, data that were not considerably homogeneous (I^2^ less than 50%) were analyzed with a fixed-effects model, whereas data that were significantly homogeneous were studied with a random-effects model.

Results

**Figure 1:**
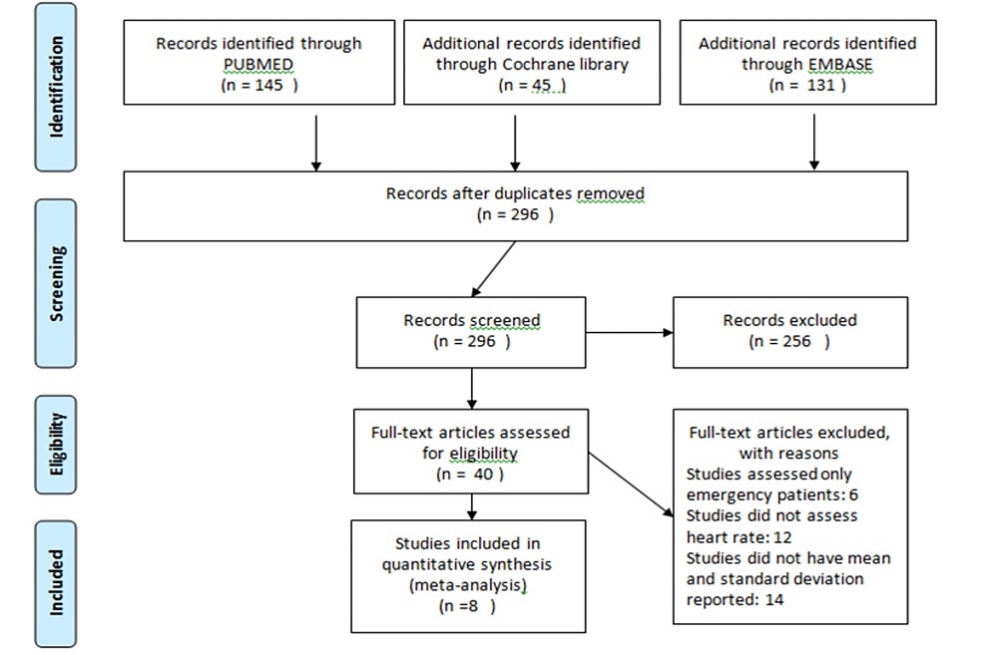
PRISMA flow chart for selection of articles in the meta-analysis PRISMA: Preferred Reporting Items for Systematic Reviews and Meta-Analyses.

**Figure 2:**
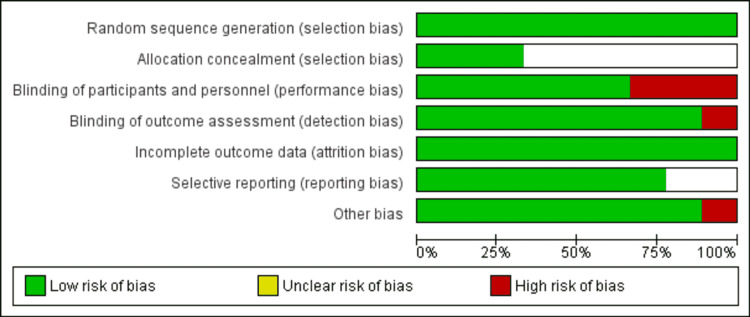
The risk of bias assessment of the included studies

Figure 1 shows the PRISMA flow chart of the current study. Overall, 321 studies were identified through the initial search. After removing 25 duplicates, the title and abstract of 296 titles were screened. Only 40 studies were eligible for the full-text screening. In this meta-analysis, eight RCTs were included, involving 548 patients (274 in the fentanyl group and 274 in the dexmedetomidine group). The characteristics of the included studies in the current meta-analysis are represented in Table 1. Only one study compared fentanyl, dexmedetomidine, and lidocaine [11]. All other studies have compared only fentanyl and dexmedetomidine [12-18]. In this meta-analysis, lidocaine was ignored, as the aim of this meta-analysis was to compare fentanyl and dexmedetomidine. Besides this, this meta-analysis will only compare the HR at one minute, five minutes, and 10 minutes after intubation between fentanyl and dexmedetomidine. All other outcomes were not considered. One study included only specific surgery patients such as laparotomy [11], neurosurgical procedure [12], and cardiac surgery [18]. All other studies included all kinds of elective surgical procedures [13-17]. Two reviewers assessed the risk of bias and it was found to be perfectly consistent, which showed that the overall quality of the study was moderate. Figure 2 shows the risk of bias plot.

**Table 1:** Characteristics of included studies Inj.: injection; IV: intravenous.

Source	Groups	Number of participants	Dose of drug	Surgical procedure
Anjum et al. (2019) [12]	D	30	Inj. dexmedetomidine 1 µg/kg IV	Elective neurosurgical procedures
	F	30	Inj. fentanyl 2 µg/kg iv	
Arif et al. (2017) [13]	D	24	Inj. dexmedetomidine 0.75 µg/kg IV	Elective surgical procedure
	F	24	Inj. fentanyl 2 µg/kg IV	
Garg et al. (2020) [14]	D	50	Inj. dexmedetomidine 1 µg/kg IV	Elective surgical procedure
	F	50	Inj. fentanyl 2 µg/kg IV	
Gauchan et al. (2019) [15]	D	30	Inj. dexmedetomidine 1 µg/kg IV	Elective surgical procedure
	F	30	Inj. fentanyl 2 µg/kg IV	
Gunalan et al. (2015) [16]	D	30	Inj. dexmedetomidine 1 µg/kg IV	Elective surgical procedure
	F	30	Inj. fentanyl 2 µg/kg IV	
Mahiswar et al. (2022) [17]	D	50	Inj. dexmedetomidine 0.5 µg/kg IV	Elective surgical procedure
	F	50	Inj. fentanyl 2 µg/kg IV	
Mahjoubifard et al. (2020) [18]	D	30	Inj. dexmedetomidine 1 µg/kg IV	Elective cardiac surgery
	F	30	Inj. fentanyl 2 µg/kg IV	
Vaswani et al. (2017) [11]	L	30	1.5 mg/kg intravenous lidocaine	
	D	30	Inj. dexmedetomidine 0.5 µg/kg IV	Elective laparotomy
	F	30	Inj. fentanyl 0.5 µg/kg IV	

HR at Baseline

**Figure 3:**
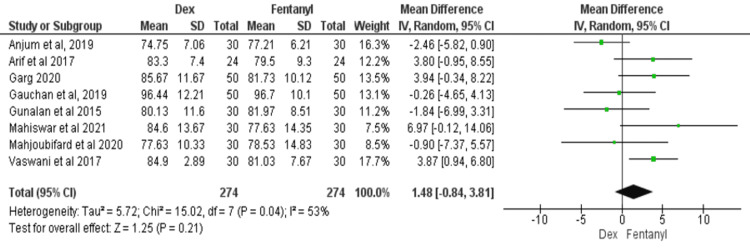
Forest plot of heart rate at baseline SD: standard deviation; Dex: dexmedetomidine. Source: [11-18].

All eight studies reported HR at baseline [11-18]. The meta-analysis results showed no significant difference in HR between the two groups at baseline (MD = 1.48; 95% CI: -0.84, 3.81; P = 0.21, I^2^ = 53%), as shown in Figure 3.

HR at One Minute After Intubation

**Figure 4:**
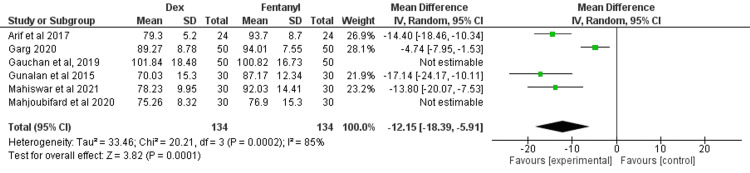
Forest plot of heart rate at one minute after intubation SD: standard deviation; Dex: dexmedetomidine. Source: [13-18].

Six studies compared the HR between dexmedetomidine and fentanyl after one minute of intubation [13-18]. There was significant heterogeneity among the results (I^2^ = 85%), and the random-effects model was used for this meta-analysis. The findings showed a significant HR difference between dexmedetomidine and fentanyl at one minute of intubation (MD = -8.46; 95% CI: -14.01, -2.92; P = 0.003), as shown in Figure 4.

HR at Five Minutes After Intubation

**Figure 5:**
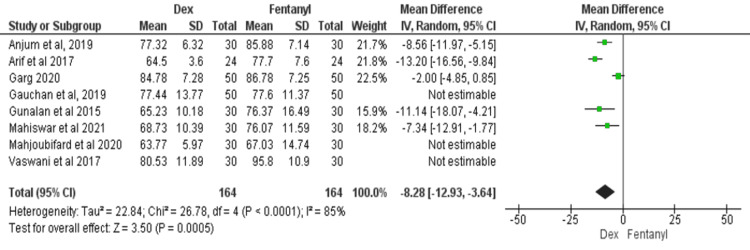
Forest plot of heart rate at five minutes after intubation SD: standard deviation; Dex: dexmedetomidine. Source: [11-18].

All eight studies compared the HR between dexmedetomidine and fentanyl after five minutes of intubation [11-18]. There was significant heterogeneity among the studies (I^2^ = 84%). For this meta-analysis, a random-effects model was used. The findings showed a significant HR difference between dexmedetomidine and fentanyl at five minutes after intubation (MD = -7.51; 95% CI: -11.42, -3.59; P = 0.001), as shown in Figure 5.

HR at 10 Minutes After Intubation

**Figure 6:**
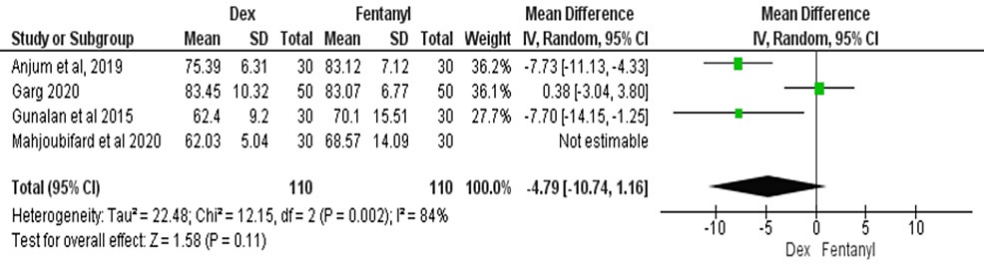
Forest plot of heart rate at 10 minutes after intubation SD: standard deviation; Dex: dexmedetomidine. Source: [12,14,16,18].

Four studies compared the HR between dexmedetomidine and fentanyl after 10 minutes of intubation [12-14,18]. There was significant heterogeneity among the studies (I^2^ = 77%). For this meta-analysis, a random-effects model was used. The findings showed a significant HR difference between dexmedetomidine and fentanyl at 10 minutes after intubation (MD = -5.15; 95% CI: -9.71, -0.59, P = 0.030), as shown in Figure 6.

Because of the significant heterogeneity in the results at all three points, a sub-group analysis was done by merging only those studies that resulted in non-significant heterogeneity. Table 2 shows the subgroup analysis, and the results are similar to the overall analysis.

**Table 2:** Subset analysis of predictive data for HR at one minute, five minutes, and 10 minutes after intubation HR: heart rate; MD: mean difference; CI: confidence interval.

HR	No. of studies	MD (95% CI)	P-value
1 minute after intubation	4 [12,14,16,17]	-12.15 (-18.39, -5.91)	0.001
5 minutes after intubation	5 [12-14,16,17]	-8.28 (-12.93, -3.64)	0.001
10 minutes after intubation	3 [12,14,16]	-4.79 (-10.74, -2.36)	0.011

Discussion

General anesthesia, comprising of four states that include immobility, analgesia, amnesia, and unconsciousness, is a drug-induced reversible situation. It also includes the physiological systems' stability, including the thermoregulatory, respiratory, cardiovascular, and autonomic systems [19]. Because endotracheal intubation is a powerful adrenergic stimulus, hypnotics are frequently used to render wakeful individuals unresponsive [20].

This meta-analysis was done to compare the efficacy of two drugs, i.e., fentanyl and dexmedetomidine, in achieving stability after intubation. Fentanyl is considered a synthetic opioid agonist phenyl pyridine derivative that has been used to reduce the hemodynamic response to intubation and laryngoscopy [9]. It also has various other benefits, including intraoperative analgesics. However, because opioids are categorized as narcotic substances, obtaining fentanyl is not without difficulty [10]. Because of this, the usage of fentanyl is regulated by national drug control policy and international treaties.

Dexmedetomidine can effectively decrease the stress response, reducing a hemodynamic response after intubation and laryngoscopy [21]. It can also increase HR stabilization at the time of surgery. It can decrease and suppress the intraocular pressure that occurs because of intubation and laryngoscopy [22]. With dexmedetomidine, analgesia and sedation can be obtained without causing hemodynamic and respiratory depression. The lack of respiratory depression is of great benefit if endotracheal intubation is difficult and fails [23].

In the current meta-analysis, the HR was significantly lower in the dexmedetomidine group as compared to the fentanyl group at one minute of intubation, five minutes of intubation, and 10 minutes of intubation. However, the level of heterogeneity was high among these outcomes, as I^2^ was more than 50% in each of the outcomes. In both groups, HR before intubation was similar. Dexmedetomidine was more efficient in preventing a rise in the hemodynamic response to the intubation and laryngoscopy as compared to fentanyl. It was due to the fact that dexmedetomidine will inhibit the release of neurotransmitters at the end of nerves, causing a reduction in levels of norepinephrine in plasma that create cardiovascular stabilization [24]. Even though less effective, fentanyl can reduce a hemodynamic response by suppressing pain cues, lowering the central sympathetic tone, and increasing vagal tone activation [24].

The current meta-analysis has certain limitations. First, it is possible that certain studies that fulfilled the inclusion criteria may have been missed in this meta-analysis. Besides this, some studies were excluded as the values of HR were not in the form of mean and SD or CI. Second, most of the studies did not determine HR at 10 minutes or later. Thus, this meta-analysis was not able to assess the long-term effect of dexmedetomidine and fentanyl after intubation. However, this question is very meaningful for the studies in the future.

## Conclusions

In the current meta-analysis, dexmedetomidine was better at preventing tachycardia following endotracheal intubation than fentanyl. HR was significantly lower at one minute, five minutes, and 10 minutes after intubation in the dexmedetomidine group as compared to the fentanyl group. HR before intubation was similar in both groups. Even though HR was lower in both groups after intubation as compared to baseline, dexmedetomidine was more efficient in preventing a rise in the hemodynamic response to the intubation as compared to fentanyl. Thus, dexmedetomidine may be recommended for better HR stability after intubation.
